# Carotid Plaque, Coronary Calcium, and Coronary Plaque: Triple Threat of Atherosclerosis in Familial Hypercholesterolemia

**DOI:** 10.1016/j.jacasi.2025.06.004

**Published:** 2025-07-22

**Authors:** Reed Mszar, Jamal S. Rana

**Affiliations:** a Department of Chronic Disease Epidemiology, Yale School of Public Health, New Haven, Connecticut, USA; b Department of Cardiology, The Permanente Medical Group, Oakland, California, USA; c Division of Research, Kaiser Permanente Northern California, Oakland, California, USA

**Keywords:** atherosclerosis, coronary artery calcium, familial hypercholesterolemia, plaque, young adults

**Figure:**
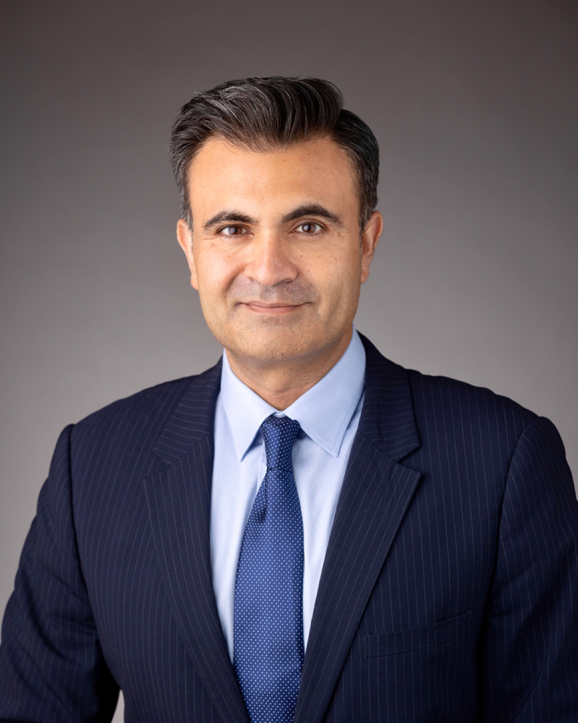

Familial hypercholesterolemia (FH) is a common genetic disorder that affects approximately 1 in 220-250 individuals and is associated with an increased risk of coronary heart disease (CHD).^1^ Despite the effectiveness of well-established lipid-lowering therapies (eg, high-intensity statins and ezetimibe) and more novel therapeutic agents (eg, PCSK9 inhibitors and inclisiran), a large proportion of individuals with FH remain undiagnosed and undertreated for their condition.^2^ Early screening, timely diagnosis, and aggressive treatment are critical to reduce the cardiovascular risks associated with FH. With an estimated 3.6-9.0 million individuals with FH living in East and Southeast Asia,^3^ there is an immense opportunity to not only reduce the risks of FH in a vast number of people, but also to enhance individuals’ quality of life and improve their health outcomes.

While those with FH broadly represent a high-risk group for premature CHD, certain individuals have a higher risk than others and not all individuals with FH ultimately experience an adverse event. As a result, increased attention has focused on personalized risk stratification strategies to assess and classify risk based on subclinical atherosclerosis, including the use of coronary computed tomography angiography (CCTA), carotid ultrasound, and coronary artery calcium (CAC) scores. Those with FH and CAC (ie, CAC > 0) are at an especially high risk of adverse events and are likely to benefit to a greater degree from an intensive approach to lipid-lowering consisting of add-on therapies to high-intensity statins.^4^^,^^5^ With an estimated 55%-58% of individuals with FH having CAC > 0,^6^^,^^7^ the use of CAC scores can guide the allocation of additional treatments and aid in shared decision-making discussions in those deemed to be at borderline or intermediate risk.^8^ Few studies, however, have examined the optimal timing for evaluating subclinical atherosclerosis across different imaging modalities in those with FH.

In this issue of *JACC: Asia*, Tada et al^9^ sought to address this evidence gap by conducting a 3-pronged assessment of subclinical atherosclerosis (ie, carotid plaque, coronary plaque, and CAC) in patients with heterozygous FH who were admitted to Kanazawa University Hospital between 2000 and 2020.^9^ Based on the Japan Atherosclerosis Society (2022) clinical criteria,^10^ 622 patients with FH were retrospectively included in the study cohort (mean age: 54 years; 49.2% male; 98.1% on statin therapy), of which 68.3% had a pathogenic FH variant. Tada et al^9^ classified patients into 3 groups by their combined atherosclerotic assessment scores (group 1: all scores = 0 [32.3%]; group 2: any score >0 to ≤ median [40.5%]; group 3: any score > median [27.2%]) and assessed risk of CHD events (defined as CHD-related death, unstable angina, myocardial infarction, or staged revascularization).

Over a median follow-up period of 13.2 years, there were 132 CHD events. Event rates of 0.3, 19.4, and 65.9 CHD events per 1,000 person-years were observed for groups 1, 2, and 3, respectively. These results are similar to those from previous studies on increasing CAC strata and risk of atherosclerotic cardiovascular disease (CVD) events.^11^, ^12^, ^13^ Notably, Tada et al^9^ also ascertained that carotid plaque, coronary plaque, and CAC, on average, develop at 17, 26, and 31 years of age in men and 30, 36, and 40 years of age in women, respectively. Based on these findings and evidence that early initiation of statins and/or combination therapy can reduce individuals’ low-density lipoprotein cholesterol (LDL-C) levels and atherosclerotic CVD risk down to that of the general population without FH,^2^ the investigators^9^ recommend CCTA screening in those with FH at 20 years of age to identify early signs of CAC and coronary plaque. Given the advantages and drawbacks of CCTA, particularly relative to the strengths of CAC scoring (eg, lower radiation exposure, easier of administration and interpretation, lower cost, wider availability), careful consideration should be given toward the exact atherosclerotic assessment strategy recommended for individuals with FH and at which age(s) would be most appropriate and evidence-based.

However, what is known is that atherosclerosis develops across the life course and, among those with FH, this process occurs earlier in life and at a more rapid rate than those in the general population. One recent study, conducted in asymptomatic individuals with genetically confirmed FH and age- and sex-matched unaffected control subjects, found that cumulative LDL-C exposure was the key driver of coronary atherosclerosis and total plaque volume in FH.^14^ These findings, which highlight the importance of early and intensive lipid-lowering treatment and the potential for subclinical atherosclerosis monitoring, can be interpreted alongside evidence that greater improvements in ideal cardiovascular health during young adulthood are independently associated with a lower risk for incident CVD in midlife.^15^

With carotid plaque being detectable earlier in life than CAC and coronary plaque in individuals with FH, it is equally important to address the role of carotid ultrasound as a low-cost, noninvasive, and safe imaging test with no radiation exposure to assess atherosclerotic plaque and/or carotid intima-media thickness (cIMT) in young individuals with FH. Observed differences in cIMT may be significant as early as 8 years of age between children with FH and their unaffected siblings.^16^ A 20-year follow-up study of statin therapy in children confirmed the long-term efficacy of early initiation of lipid-lowering therapy by reporting a slowed progression of cIMT and decreased risk of CVD in adulthood.^17^ Among children (aged 8-18 years) with FH, different treatment algorithms have been proposed based on cIMT percentile (<75th vs ≥75th) and plaque presence/absence^5^; however, the lack of a standardized measurement of plaque burden, subjective analyses, and heterogenous carotid plaque phenotypes may limit its broader use in clinical practice.

It is important to note that the current findings are generalizable to individuals who were admitted to a single academic hospital in Japan and may not apply broadly to all individuals with FH in other regions, including areas with lower diagnosis rates and reduced health care access. Future prospective and multicenter studies are needed to confirm these results, including the prognostic value, feasibility, and cost-effectiveness of the triple assessment of atherosclerosis in FH. Moreover, whereas previous evidence suggests that individuals with CAC = 0 should receive follow-up CAC testing in 3-7 years depending on demographic and clinical characteristics,^18^ the “warranty period” of repeat testing in FH with an absence of CAC has yet to be thoroughly investigated. This point is particularly pertinent given that a CAC score of 0 among younger individuals with FH does not necessarily preclude the presence of subclinical atherosclerosis. Pooled data from multiple international cohorts revealed that nearly 1 in 5 individuals with FH and CAC = 0 had nonobstructive coronary artery disease on CCTA,^6^ which may provide compelling rationale for a combination approach to atherosclerosis assessment.

In the era of precision medicine, incorporating polygenic risk scoring (PRS) can also play an important role in younger individuals to augment current approaches to risk stratification and enhance primary prevention strategies leveraging both lifestyle and pharmacologic interventions. In the context of FH, this is particularly relevant given that approximately 20%-30% of clinical FH cases are attributable to polygenic hypercholesterolemia,^19^ which is associated with an increased risk of CVD events compared to those with hypercholesterolemia and no identifiable genetic cause.^20^ One study showed that integrating a PRS had the largest effect with early onset cases of CHD.^21^ Importantly, even in those with a high genetic risk, comprehensively improving lifestyle factors can lead to significant reductions in CHD risk.^22^ With recent studies and clinical recommendations underscoring the role of PRS to provide additional prognostic information in high-risk groups,^23^^,^^24^ this tool can be leveraged to both improve risk reclassification for incident CHD and its communication can promote healthy lifestyle factors among those deemed to derive the greatest benefit.^25^ Additionally, with a large portion of contemporary training data deriving from individuals of European ancestry, broadening the representation of non-European populations through developing and sustaining new cohorts, such as Biobank Japan,^26^ will improve the generalizability of these risk scores to other at-risk groups. In the Asia-Pacific region, physician knowledge of FH along with awareness of clinical guidelines and available services in the region, have been shown to be suboptimal and represent significant barriers to care.^27^^,^^28^ Addressing these factors in tandem with clinicians’ knowledge and patients’ understanding of PRS can work to collectively improve the quality of FH care for affected individuals.

Engaging in comprehensive risk assessment, stratification, and management in early adulthood, particularly in high-risk groups (ie, young adults with FH), has the potential to drastically change the landscape of CVD prevention worldwide.^29^ Achieving these goals will ultimately require a multipronged approach to increasing patient and provider awareness of FH, improving access to effective pharmacotherapies, and initiating subclinical atherosclerosis assessment early and often across individuals’ continuum of care. Furthermore, fostering multinational partnerships throughout the Asia-Pacific region; enhancing current education and training programs on FH; increasing research initiatives and funding opportunities to develop high-quality and generalizable data; and improving access to affordable and effective imaging technologies across low-, middle-, and high-income countries and rural areas, represent key steps in reducing the burden of CVD among high-risk individuals with FH throughout Asia.

## Funding Support and Author Disclosures

The authors have reported that they have no relationships relevant to the contents of this paper to disclose.
